# Targeting Reduced Glutathione (GSH) to Promote Metabolic Health: Insights on the Role of Bioactive-Rich Foods and Fasting Protocols

**DOI:** 10.3390/ijms27146400

**Published:** 2026-07-18

**Authors:** Periklis Vardakas, Zoi Skaperda, Paraskevi Maria Nechalioti, Sotiria Makri, Anastasia Patouna, Maria Gkasdrogka, Thomas Karampatzakis, Kyriaki Kroustalli, Georgios Papageorgiou, Evanthia Angeli, Dimitrios Foulos, Fotios Tekos, Demetrios Kouretas

**Affiliations:** 1 Laboratory of Animal Physiology, Department of Biochemistry & Biotechnology, University of Thessaly, 41500 Larissa, Greece; pevardakas@uth.gr (P.V.); zoskaper@uth.gr (Z.S.); pnechalioti@uth.gr (P.M.N.); somak@uth.gr (S.M.); anpatouna@uth.gr (A.P.); mgkasdrogka@uth.gr (M.G.); tkarampatzakis@uth.gr (T.K.); kkroustalli@uth.gr (K.K.); 2 ProGnosis Biotech S.A., 41335 Larissa, Greece

**Keywords:** metabolic health, metabolic dysfunction, redox homeostasis, reduced glutathione, oxidative stress, chronic diseases, bioactive compounds, fasting

## Abstract

The ever-increasing disparity between lifespan and healthspan represents a challenging global issue, with metabolic dysregulation playing a central role in the initiation and progression of chronic non-communicable diseases (NCDs). This review highlights the importance of maintaining optimal redox homeostasis, with particular emphasis on reduced glutathione (GSH), for preserving metabolic health during aging. GSH participates in several physiological processes, including antioxidant defense, xenobiotic detoxification, redox signaling, and metabolic regulation. Diminished GSH levels are consistently reported in obesity, insulin resistance, type 2 diabetes mellitus, non-alcoholic fatty liver, and cardiovascular diseases. Current evidence from human clinical studies indicates that foods rich in bioactive constituents can enhance GSH levels and stimulate GSH-dependent enzyme activity, with the Nrf2/Are signaling pathway being a central mechanistic link. Fasting may promote adaptive redox responses by inducing mild oxidative stress and activating the same molecular mechanism, although the effects on GSH-related antioxidant mechanisms remain heterogeneous across fasting protocols and study populations. Altogether, the available clinical evidence suggests that these nutritional and lifestyle interventions exhibit more consistent beneficial effects in individuals characterized by increased oxidative burden and underlying metabolic dysfunction. Interindividual differences in GSH responses further underscore the need for targeted, tailor-made approaches that account for genetic, epigenetic, and lifestyle factors. Collectively, targeting GSH homeostasis through nutritional and lifestyle interventions represents a promising strategy for improving metabolic health and may further contribute to healthy aging, positioning redox biology at the forefront of aging research and NCD prevention.

## 1. The Lifespan–Healthspan Gap: The Role of Metabolic Health

### 1.1. Epidemiological Burden and Public Health Implications

Life expectancy at birth (LEB), defined as the average number of years a newborn is expected to live under the assumption that current age-specific mortality rates remain constant throughout their lifetime [1], has increased markedly over recent decades, expanding by approximately 30 years in economically prosperous nations [2]. This progress has been driven primarily by improvements in food availability and quality, sanitation, healthcare systems, and advances in biomedical science [2,3]. As a matter of fact, World Health Organization (WHO) data (last updated in 2024) report a current global LEB of about 71 years and a regional average of roughly 76 years in Europe for both sexes [4]. On the contrary, healthy life expectancy at birth (HALE), referring to the average number of years a newborn is expected to live in good health and with functionality, is estimated at approximately 61.9 years globally and around 66 years in Europe for both sexes [5]. The resulting gap between LEB and HALE has created a widening deficit of 9–10 years, during which older individuals experience a significant deterioration in life quality, frequently characterized by pronounced fatigue, metabolic dysfunction, and the burden of chronic non-communicable diseases (NCDs) [6].

The discrepancy between these two key indicators used to measure and benchmark population health and well-being [7] underscores a fundamental epidemiological principle: more life years do not automatically translate into additional quality-adjusted life years. Furthermore, it reveals a public health challenge, as nearly a decade of later life is spent in suboptimal health, exerting substantial pressure on health systems, diminishing economic productivity, and affecting public welfare [8]. The magnitude of this global crisis is further stressed by striking epidemiological data. More specifically, in 2023, approximately 1.5 billion individuals worldwide were affected by metabolic disorders [9], while NCDs accounted for roughly 74% of all global deaths [10]. Metabolic dysregulation represents the main biological driver of NCDs [11], which have emerged as a persistent and silent pandemic, jeopardizing the United Nations’ 2030 Agenda for Sustainable Development target of reducing premature mortality from NCDs by one-third by 2030 [12].

### 1.2. Metabolic Health as a Central Pillar of Healthy Aging and Mechanistic Foundations

These observations indicate that the historical success in prolonging lifespan has come at a considerable cost, as many formerly fatal diseases have been transformed into manageable chronic conditions [13]. Consequently, there is a growing consensus that the central challenge of contemporary aging research lies in extending healthspan, ensuring that additional life years are lived with vitality and functional independence [14]. Achieving this objective requires a multidimensional strategy that integrates primary prevention with public health policies designed to reduce morbidity and improve quality of life in later years. Within this framework, the optimization of metabolic health emerges as a cornerstone of healthy aging, considering its pivotal role in maintaining physiological homeostasis and preventing the onset and progression of NCDs [15].

Metabolic health refers to the proper functioning of metabolic processes that regulate energy balance, glucose homeostasis, lipid metabolism, insulin sensitivity, blood pressure, and body composition [16,17]. It is shaped by a complex interplay between biological factors, including age, sex, and genetic predisposition; environmental factors, including exposure to pollutants and socio-economic status; and lifestyle factors, including dietary patterns, physical activity, sleep quality, and stress [18]. The benefits of a balanced diet, regular moderate exercise, adequate sleep, and effective stress management are well established in maintaining and fine-tuning metabolic homeostasis [19,20]. This is particularly important because disruption of physiological metabolic regulation increases the risk of metabolic dysfunction, which is strongly associated with insulin resistance, type 2 diabetes mellitus, dyslipidemia, hypertension, obesity, and cardiovascular diseases [21,22].

At the mechanistic level, these conditions are commonly related to chronic low-grade inflammation, oxidative stress, and impaired mitochondrial function [23]. Collectively, these processes promote cellular damage and disrupt the delicate redox balance that dictates metabolic signaling [24]. Mitochondrial dysfunction may amplify the production of reactive species, thus altering crucial redox-sensitive pathways involved in nutrient sensing and insulin signaling [25]. This shift from physiological to pathological redox signaling emerges as a key driver of metabolic health decline, converting localized cellular stress into broader metabolic dysfunction [24].

## 2. Redox Homeostasis: The Missing Link Between Aging and Metabolic Dysfunction

### 2.1. The Oxygen Paradox and Antioxidant Defense Systems

Redox status describes the dynamic equilibrium between oxidants and antioxidants that sustains cellular homeostasis and supports normal biological function [26]. In aerobic life, this balance is of paramount importance due to the ambiguous role of oxygen, a classic biological concept known as the “Oxygen Paradox” [27]. According to this, oxygen is indispensable for mitochondrial bioenergetics and ATP synthesis; however, it also contributes to the generation of reactive oxygen species (ROS), chemically reactive moieties capable of inducing oxidative damage when produced in excess during metabolism [28].

To counteract the accumulation of oxidizing agents, aerobic organisms have developed effective defense systems, in which antioxidants play a central role [29]. Antioxidants are broadly defined as any substance that delays, prevents or removes oxidative damage to a target molecule [30]. This definition includes endogenous low-molecular-weight compounds, such as reduced glutathione, uric acid, bilirubin, coenzyme Q10, and lipoic acid, as well as enzymatic systems, including catalase (CAT), superoxide dismutase (SOD), glutathione peroxidase (GPx), and glutathione reductase (GR) [31,32]. Furthermore, it encompasses exogenous compounds of dietary origin, including polyphenols, carotenoids, vitamins A, C, and E, and trace elements, such as selenium, manganese, and zinc [33]. Accordingly, antioxidants constitute a highly heterogeneous group of compounds with distinct sources, chemical structures, solubilities, mechanisms and sites of action, and biological effects [34,35].

The interplay between endogenous and exogenous antioxidants within the human antioxidant arsenal is highly intricate and represents an issue of particular interest in Redox Biology, especially in efforts to prevent and ameliorate NCDs and promote metabolic health [36]. The maintenance of cellular redox homeostasis relies on the coordinated action of these antioxidant systems, as well as on their ability to preserve a critical amount of oxidants, which are responsible for various physiological processes in the human organism, such as immune defense, wound healing, regulation of vascular tone, and cell signaling, communication, and survival [37,38,39]. It is worth noting that the precise regulation of redox homeostasis is a critical determinant of cell fate, as redox balance affects most cellular functions and responses, including viability, proliferation, differentiation, metabolism, and death [40].

### 2.2. Oxidative Stress: From Eustress to Distress and NCD Pathophysiology

Nevertheless, the maintenance of redox homeostasis is an ongoing challenge. The disruption of the fine balance between oxidants and antioxidants in favor of oxidants causes oxidative stress, a phenomenon associated with disturbances in redox signaling and molecular damage [41]. Interestingly, oxidative stress is two-sided, being both beneficial and detrimental depending on its intensity, duration, and context, which indicates that deviations from redox equilibrium are not consistently harmful [42]. At low levels, termed “oxidative eustress”, it is essential for physiological redox regulation and signaling, thus contributing to the maintenance of cellular homeostasis. In contrast, at elevated and persistent levels, termed as “oxidative distress”, it disrupts redox signaling and promotes oxidative damage to critical biomolecules [43].

Oxidative stress can adversely affect lipids, proteins, and nucleic acids, thereby compromising cell viability. The oxidative degradation of lipids disturbs the integrity of biological membranes [44]. Proteins are also susceptible to oxidation, undergoing conformational changes that may impair their structural or functional activities [45]. In addition, oxidative burden can cause toxic insults to DNA, related to the loss or oxidation of nitrogenous bases and the formation of strand breaks [46]. Chronic exposure to oxidative stress may lead to a subclinical state of progressive cellular damage that accumulates over time, driving tissue remodeling and resulting in selective organ dysfunction and disease [47]. Consistent with this, high levels of oxidative stress have been documented in several NCDs, for instance atherosclerosis, Alzheimer’s disease, Parkinson’s disease, type 2 diabetes mellitus, and various cancer types [48]. Oxidative stress is not only implicated in the onset of NCDs, but it is also a critical determinant of their progression [49]. Therefore, monitoring clinically relevant markers of oxidative stress may enable the early identification of redox disturbances prior to the manifestation of clinical symptoms, supporting the prevention of metabolic dysfunction and the development of NCDs [47].

## 3. Glutathione: The Primary Redox Buffer

### 3.1. Biosynthesis and Distribution

Glutathione is the most abundant and important low-molecular-weight thiol in mammalian cells, with intracellular concentrations typically ranging from 0.1 to 10 mM in the cytosol and generally remaining within 1–2 mM in most cell types [50]. The case is different for hepatocytes, where levels may reach around 10 mM, reflecting both the central role of the liver in glutathione biosynthesis and the significant contribution of glutathione to xenobiotic detoxification [51].

With regard to subcellular distribution, approximately 80–85% of total glutathione is localized in the cytosol, about 15% is found in mitochondria, and the remaining fraction is distributed between the endoplasmic reticulum and the nucleus [50,52]. Glutathione exists in cells in two readily interchangeable forms: the reduced form (GSH), which is biologically active because of its sulfhydryl (-SH) group, and the oxidized form, glutathione disulfide (GSSG), generated through the formation of a disulfide bond between two GSH molecules [53]. Under normal conditions, 98–99% of the intracellular glutathione pool is maintained in its reduced state [54], and the GSH/GSSG ratio serves as a sensitive biomarker of oxidative stress [55]. In normal, unstressed cells, the ratio typically exceeds 100:1, whereas under oxidative stress it may drop to 10:1 or even lower [56].

Structurally, GSH is a tripeptide comprising the non-essential amino acids cysteine, glycine, and glutamate [57]. Its de novo biosynthesis occurs in the cytosol via a two-step process, catalyzed by glutamate–cysteine ligase (GCL) and glutathione synthase (GS) [58]. To be more specific, in the first step, GCL catalyzes the formation of the dipeptide γ-glutamyl-cysteine through a peptide bond between the γ-carboxyl group of glutamate and the amino group of cysteine. Subsequently, GS catalyzes the addition of glycine to the dipeptide, resulting in the generation of GSH [51,57]. The overall rate of GSH biosynthesis is regulated by various key factors, including the intracellular availability of substrates, particularly L-cysteine, the expression level and stoichiometric balance of the two GCL subunits, namely the catalytic subunit (GCLC) and the modifier subunit (GCLM), and the degree to which GCL activity is suppressed by feedback from GSH [59].

### 3.2. Biological Roles and Cellular Homeostasis

GSH exerts its antioxidant activity through both direct and indirect mechanisms. The redox-active thiol (-SH) group of its cysteine residue enables GSH to participate in thiol-mediated redox reactions, serving as a critical intracellular source of reducing equivalents [60]. GSH directly neutralizes reactive oxygen species (ROS) and reactive nitrogen species (RNS) via two distinct molecular mechanisms, hydrogen atom transfer (HAT) and single electron transfer (SET), the predominance of which depends on the chemical nature and redox potential of the oxidant [54,61]. HAT is particularly relevant for highly reactive oxygen-centered radicals, such as hydroxyl (^•^OH) and lipid peroxyl (LOO^•^) radicals. In this pathway, GSH donates a hydrogen atom through the homolytic cleavage of the cysteine sulfur–hydrogen bond, neutralizing the corresponding radical and yielding a less reactive glutathione thiyl radical (GS^•^) [62,63]. Contrariwise, SET is favored with strongly electrophilic radicals, radical cations, transition metal ions, and certain RNS. Herein, GSH transfers a single valence electron from the non-bonding lone pair of the nucleophilic cysteine sulfur atom to the oxidant, thus reducing it and generating unstable glutathione-derived radical intermediates that rapidly transform into GS^•^ [64,65]. Once formed, GS^•^ is rapidly converted to GSSG, with radical–radical recombination predominating especially when GS^•^ concentration is relatively high. Together, these discrete, yet complementary, pathways allow GSH to directly detoxify a broad spectrum of reactive species, with recent evidence indicating that HAT constitutes the primary mechanism underlying its direct radical-scavenging activity [66].

In addition, GSH serves as a substrate for glutathione peroxidases (GPxs), an eight-member family of selenoenzymes that catalyze the detoxification of hydrogen peroxide and lipid hydroperoxides [67]. GPxs employ a selenocysteine residue at their active site to transfer two electrons to the peroxide substrate, converting it to water or the corresponding alcohol [68]. During this reaction, the selenol (Se-H) is oxidized to selenic acid (Se-OH) or higher oxidation states [69,70]. Then, two GSH molecules sequentially donate electrons to reduce oxidized selenium back to its selenol form, restoring GPx enzymatic activity and producing GSSG through the conjugation of two oxidized GSH molecules [71].

To preserve the redox cycle, GSH is regenerated from GSSG by glutathione reductase (GR) activity [72]. In this catalytic process, nicotinamide adenine dinucleotide phosphate (NADPH) acts as a hydride (H^−^) donor to the enzyme-bound flavin adenine dinucleotide (FAD), reducing it to FADH_2_ while simultaneously being oxidized to NADP^+^ [73]. Subsequently, FADH_2_ transfers two electrons to the disulfide bond of GSSG, whereas a proton from the surrounding environment provides the additional hydrogen required for the formation of two functional sulfhydryl groups, yielding two GSH molecules [74]. Alternatively, GSH is exported to the extracellular space and cleaved into its constituent amino acids by γ-glutamyl transpeptidase (GGT), which can then be reutilized for de novo biosynthesis [72].

Beyond its fundamental role in preventing oxidative stress and maintaining redox balance, GSH also contributes to the regulation of a broad range of physiological processes, including redox signaling, cellular metabolism, and biosynthetic homeostasis [59]. In addition to its established function in antioxidant defense, GSH participates in the preservation of protein thiol redox status, the protection of cysteine residues from irreversible oxidation and aberrant cross-linking, and ascorbate metabolism [67,75]. Furthermore, GSH plays an important role in xenobiotic detoxification, radioprotection, DNA repair, protein folding, and the degradation of proteins containing disulfide bonds [76,77,78]. Moreover, it is involved in the control of cell cycle progression, proliferation, differentiation, communication, apoptosis, ferroptosis, energy metabolism, and immunologic reactions [59,79,80]. Collectively, these functions underscore the central and multifaceted role of GSH in preserving cellular integrity and supporting physiological cellular homeostasis.

### 3.3. Blood Dynamics and Pathological Alterations in Metabolic NCDs

In blood, GSH concentration varies considerably between the cellular and liquid compartments, corresponding to red blood cells and plasma, respectively. In healthy individuals, erythrocytes typically contain GSH at levels ranging from 0.4 to 3.0 mM, reflecting their capacity to synthesize and store GSH [81], whereas plasma levels are substantially lower, generally ranging from 1.0 to 6.0 μM, largely associated with liver production and systemic release [82].

Alterations in blood GSH levels are widely recognized as an important and sensitive indicator of redox perturbations [59]. In general, elevated GSH levels may indicate an adaptive cellular response, aiming to reinforce antioxidant defenses and protect against the detrimental effects of oxidative stress on cellular integrity [83]. However, in many chronic diseases, frequently characterized by excessive and persistent oxidative stress, the intracellular GSH pools are instead depleted [84]. This may result from a combination of factors, including high consumption during the scavenging of ROS and RNS, impaired de novo biosynthesis because of substrate limitation, reduced catalytic activity of key biosynthetic or regenerating enzymes, and increased utilization in detoxification pathways [83]. Such declines compromise redox buffering capacity and may further exacerbate oxidative damage, thereby contributing to disease progression.

NCDs associated with metabolic dysregulation, such as obesity, insulin resistance, type 2 diabetes mellitus, and cardiovascular diseases, share overlapping pathophysiological mechanisms, with oxidative stress driven by mitochondrial dysfunction representing a central contributor [85]. Obesity is related to an oversupply of glucose and free fatty acids, thereby perturbing mitochondrial substrate oxidation and disrupting energy metabolism [86]. This promotes the accumulation of acetyl coenzyme A (coA), impairs normal mitochondrial function, and enhances electron leakage from the electron transport chain, which reacts with molecular oxygen to increase intracellular ROS production [86]. These effects are further amplified by complications within the adipose tissue microenvironment and chronic low-grade inflammation, thus aggravating the oxidative burden [87]. Notably, erythrocyte GSH levels appear to be lower in obese individuals [88], while higher baseline concentrations have been interestingly associated with a better response to dietary interventions for weight loss [89].

Type 2 diabetes mellitus primarily arises from the interaction between impaired pancreatic insulin secretion and insulin resistance in peripheral tissues, altogether leading to persistent hyperglycemia [90]. In turn, chronic hyperglycemia induces oxidative stress, which disturbs insulin signaling pathways and compromises pancreatic β-cell function, further exacerbating insulin resistance [91]. Reduced GSH levels have been reported in insulin resistance and dysfunction of pancreatic β-cells, and they are also consistently observed in patients with type 2 diabetes mellitus [84,91].

Cardiovascular diseases, representing the leading cause of mortality and morbidity globally, are closely related to oxidative stress, which contributes both to their onset and progression [92]. The excessive generation of ROS within the vascular system promotes endothelial dysfunction and inflammation, reduces nitric oxide bioavailability, and impairs vasodilatory capacity [93]. Collectively, these alterations disrupt vascular tone, induce the formation of atherosclerotic plaques, contribute to myocardial injury, and drive adverse cardiac remodeling [94,95]. It is worth noting that GSH deficiency has been reported in heart failure, coronary artery disease, and myocardial infarction, supporting the critical role of GSH homeostasis in cardiovascular pathology [96,97,98,99].

## 4. Dietary and Lifestyle Interventions to Enhance Glutathione: Evidence from Human Trials

### 4.1. Bioactive-Rich Foods and Their Effects on GSH Metabolism

In recent years, scientific interest has shifted towards food products that, in addition to supplying energy and providing essential macro- and micronutrients, may further exert beneficial effects on human health and well-being. A substantial body of evidence demonstrates that a wide range of plant-derived foods are particularly rich in bioactive constituents, such as polyphenols and flavonoids, carotenoids, terpenoids, alkaloids, and organosulfur compounds, with antioxidant, anti-inflammatory, antimicrobial, and even antitumor activities, thus extending their biological relevance beyond conventional nutrition [100,101,102]. These phytochemicals can modulate redox-sensitive pathways, influence inflammatory cascades, and support endogenous defense mechanisms, rendering them promising non-pharmacological candidates for reinforcing redox homeostasis, improving metabolic health, and reducing the risk of NCDs [103]. Their protective effects in the human organism are exerted primarily by acting as effective modulators of the transcription factor nuclear factor erythroid-derived 2-like 2 (Nrf2)/antioxidant response element (ARE) pathway [104,105,106]. Indeed, following ingestion, most of these exogenous antioxidants are extensively metabolized by the gut microbiota before reaching systemic circulation, resulting in poor bioavailability [107,108]. Consequently, their systemic concentrations are typically extremely low and, therefore, incapable of directly scavenging ROS [109]. This signifies that the main mechanism by which phytochemicals exert their antioxidant activities in vivo is probably the activation of Nrf2 [110].

This review places special emphasis on various food products that interact with GSH metabolism, including those that preserve intracellular GSH pools, facilitate GSH regeneration, or enhance glutathione-dependent antioxidant systems. Particular attention is given to pilot studies and clinical trials in human subjects, as their findings are more directly translatable than those obtained from in vitro studies or preclinical models.

#### 4.1.1. Green Tea

Green tea is widely recognized as a rich source of bioactive compounds, most notably catechins, among which epigallocatechin-3-gallate (EGCG) is the main polyphenol contributing to its antioxidant activity [111]. Evidence from human intervention studies demonstrates that the impact of green tea supplementation on GSH metabolism is modulated by the underlying metabolic and redox status of the target population. As a case in point, in a randomized pilot study, 16 healthy males (22–23 years) following a 6-week CrossFit training program were equally assigned to receive either a placebo or green tea extract (500 mg/day) and completed two exercise trials, one before and one after the intervention [112]. The results revealed no significant changes in whole blood GSH concentration or erythrocyte GPx and GR activities [112]. Nevertheless, green tea significantly increased ferric reducing antioxidant power (a) and reduced lipid peroxidation byproducts in plasma compared with placebo [112]. These findings suggest that in healthy and physically active individuals, green tea primarily improves overall antioxidant capacity and decreases exercise-induced oxidative damage rather than directly regulating GSH metabolism [112].

On the contrary, more prominent effects have been reported in populations with metabolic complications. In a randomized controlled trial involving 35 obese individuals with metabolic syndrome (8 males and 27 females; 25–63 years), daily consumption of green tea (4 cups/day) for 8 weeks caused a significant increase in whole blood GSH concentration and plasma antioxidant capacity in comparison with the control group (4 cups of water/day). These results indicate that regular green tea intake can enhance antioxidant defenses in individuals characterized by chronic oxidative stress, potentially through the elevation of GSH pools, and thereby support a protective role in metabolic dysfunction [113]. Although serum GPx activity was also assessed, no significant differences were observed between the two groups, suggesting that the intervention primarily increased GSH availability rather than GSH-dependent enzymatic activity [113]. Consistent with these findings, an ex vivo study demonstrated that green tea (-) epicatechin significantly increased erythrocyte GSH concentration and decreased markers of lipid peroxidation and protein carbonylation in a concentration-dependent manner in blood samples from both hypertensive and healthy individuals, with more pronounced effects in hypertensive subjects [114]. These results support the notion that catechin-rich green tea particularly benefits individuals with a higher oxidative burden by improving GSH status and reducing oxidative damage [114].

#### 4.1.2. Tomato Juice

Tomato juice is an excellent source of lycopene, a potent antioxidant with health-promoting properties [115]. Nevertheless, human intervention studies investigating its impact on GSH-related outcomes have yielded contradictory findings, suggesting that the response to tomato juice may be contingent upon the health status and baseline redox profile of the target population. Specifically, in 25 healthy male early adolescents (10–11 years), an 18-day supplementation with tomato juice (240 g/day) significantly increased plasma lycopene concentrations, confirming the efficient absorption and high bioavailability of tomato-derived carotenoids; yet, it did not cause any significant alterations in whole blood GSH concentration [116]. Similarly, in a cohort of 15 ultra-marathon runners (13 males and 2 females; 36–53 years), a 2-month supplementation with tomato juice, provided in amounts isocaloric with their customary carbohydrate supplement, did not modify erythrocyte GSH concentration, despite improving the overall redox profile, as reflected by reduced lipid peroxidation and protein carbonylation [117]. Collectively, these studies suggest that in healthy populations, tomato juice enhances antioxidant capacity and attenuates lifestyle- or exercise-induced oxidative stress; however, it does so without substantially engaging GSH-related pathways.

The case appears to be different when examining the effects of tomato juice in populations with metabolic impairments. More elaborately, in a randomized controlled trial, enrolling 64 overweight or obese female individuals (20–30 years), the 20-day consumption of tomato juice (330 mL/day) resulted in a significant increase in erythrocyte GPx activity, accompanied by elevated total antioxidant capacity and decreased lipid peroxidation byproducts in plasma, compared to both the baseline values and the control group, indicating an enhancement of GSH-dependent antioxidant defense following short-term tomato juice intake [118]. Furthermore, in a randomized, cross-over clinical trial involving 61 obese young individuals (33 males and 28 females; 8–13 years) with non-alcoholic fatty liver disease (NAFLD), participants followed either a calorie-restricted regimen (1200 kcal/day) alone or in combination with lycopene-rich tomato juice (100 mL/day). The 60-day supplementation with tomato juice increased serum GSH concentration, decreased serum GSSG concentration, and therefore raised the GSH/GSSG ratio, reflecting a significant improvement in systemic redox status [119]. These changes were accompanied by reductions in pro-inflammatory indicators, favorable modulation of the adipokine profile, and amelioration of hepatic steatosis, suggesting that tomato juice, particularly when combined with calorie restriction, may enhance GSH homeostasis and attenuate oxidative stress and inflammation in individuals with metabolic dysfunction [119].

#### 4.1.3. Pomegranate Juice

Pomegranate juice is particularly rich in bioactive phytochemicals, including polyphenols and anthocyanins, and its health benefits are well documented [120]. In a clinical study conducted in 13 elderly individuals (10 males and 3 females; >60 years), the daily consumption of pomegranate juice (250 mL) for 4 weeks significantly increased plasma GPx activity compared to the baseline levels, although plasma GSH concentration was not affected [121]. Despite the absence of changes in GSH levels, the intervention elevated CAT activity and FRAP levels and reduced lipid peroxidation and protein carbonylation in plasma, indicating an overall improvement in systemic redox status [121]. A different pattern emerged in a pilot study, involving 14 healthy individuals (8 males and 6 females; 30–37 years), wherein the daily consumption of pomegranate juice (500 mL) for 15 days produced a significant increase in erythrocyte GSH concentration compared to the baseline levels, an effect also accompanied by decreased protein carbonylation in plasma, thus reflecting an enhancement of blood redox homeostasis [122]. Further evidence for the GSH-modulatory effects of pomegranate juice originates from a pilot study of 9 elite male weightlifters (20–22 years) who performed training sessions after supplementation with either a placebo or pomegranate juice [123]. Both the placebo and pomegranate juice were administered at 250 mL three times daily, during the 48 h preceding each training session, with an additional 500 mL consumed 60 min before physical activity [123]. Under these conditions, pomegranate juice enhanced plasma GPx activity shortly (3 min) after the completion of the training session and accelerated the return to baseline levels after 48 h and 10 days of recovery, highlighting its capacity to strengthen GSH-related antioxidant defenses in response to intense exercise [123]. Although these findings demonstrate clear antioxidant efficacy, the relatively high acute dose (1.25 L), consumed within a short time window, raises questions as regards the long-term sustainability of, and adherence to, such a regimen, which should be evaluated in future studies.

Beyond healthy populations, pomegranate juice has also exhibited promising effects in individuals suffering from chronic degenerative conditions. Specifically, in a randomized, parallel-group clinical trial involving 39 individuals with osteoarthritis, participants were assigned either to an intervention group (2 males and 17 females; 47–67 years) consuming pomegranate juice (200 mL/daily) or to a control group (2 males and 17 females; 42–66 years) maintaining their typical lifestyle [124]. Following 6 weeks, the consumption of pomegranate juice significantly increased serum GPx activity relative to baseline levels, suggesting an upregulation of GSH-dependent antioxidant defenses [124]. This finding was accompanied by a significant reduction in serum matrix metalloproteinase 13 (MMP-13), a key enzyme implicated in cartilage degradation, compared to the control group at the end of the intervention [124]. Altogether, these data support an amelioration of oxidative stress and tissue degeneration through the reinforcement of the antioxidant arsenal.

#### 4.1.4. Grape-Derived Products

Grape juice is also considered a major source of bioactive polyphenolic constituents, including hydroxycinnamic acids, anthocyanins, and stilbenes [125]. In a randomized, controlled, crossover trial, 30 healthy individuals (9 males and 21 females; 20–38 years and 21–36 years, respectively) were randomly assigned to one of the three groups: (a) an intervention group consuming conventional tropical grape juice (400 mL/daily), (b) a second intervention group consuming organic tropical grape juice (400 mL/daily), or (c) a control group consuming water (400 mL/daily) [126]. Blood samples were collected prior to the supplementation and 1 h after beverage ingestion, and this procedure was repeated after a 14-day washout period with the administration of a different test beverage [126]. The findings revealed a significant increase in erythrocyte GSH concentration and GPx activity in both the conventional and organic tropical juice groups, compared to the baseline values and the control group [126]. Additional data supporting a role of grape-derived constituents in regulating GSH status were provided by a randomized, double-blind, placebo-controlled clinical trial, in which 40 female volleyball players (~15–29 years) received either grape seed extract (300 mg/twice daily) or a placebo for 8 weeks [127]. According to the results, grape seed extract supplementation resulted in a significant increase in plasma GSH concentration, while decreasing serum malondialdehyde (MDA) levels [127]. These results were associated with reduced serum insulin and improved insulin resistance, highlighting the close interplay between redox homeostasis and metabolic regulation [127].

The case was different in a randomized controlled intervention study evaluating the impact of a 6-week supplementation with an alcohol-free red grape skin polyphenolic extract (390 mg, three times daily) in 14 healthy male physical education students (21–22 years) undergoing interval swimming training [128]. Participants were allocated to either a supplementation group (*n* = 9) or a control group (*n* = 5), and blood antioxidant parameters were assessed before and after exercise [128]. Despite the high polyphenol content of the extract, supplementation did not significantly modify erythrocyte GSH concentration or erythrocyte GPx and GR activities in comparison with baseline values or the control group [128]. Similarly, no significant effects were observed on SOD and CAT activities or plasma total antioxidant status (TAS) [128]. Nevertheless, the extract significantly reduced exercise-induced creatine kinase release, suggesting attenuation of skeletal muscle damage, and improved exercise performance [128].

#### 4.1.5. Brazil Nuts

Two studies have investigated the impact of Brazil nuts, a particularly rich source of selenium, on GSH-related enzymes in populations with underlying health conditions. In particular, a prospective intervention study in 81 patients (55 males and 26 females; ~37–67 years) undergoing hemodialysis showed that the daily consumption of one Brazilian nut for 3 months resulted in a significant elevation in erythrocyte and plasma selenium levels, as well as erythrocyte GPx activity, with all participants achieving values within the normal reference range following supplementation [129]. In line with these findings, another prospective intervention study demonstrated that the daily consumption of one Brazilian nut daily (~290 μg selenium/daily) for 8 weeks significantly increased erythrocyte GPx activity in 37 severely obese females (28–41 years) [130]. This response was accompanied by elevations in erythrocyte and plasma selenium concentrations, higher HDL-cholesterol levels, and improved cardiovascular risk indicators [130]. Collectively, these findings confirm that regular Brazilian nut consumption can enhance GSH-dependent pathways through improved selenium bioavailability, while also providing cardiometabolic benefits in the case of obesity.

#### 4.1.6. Functional Bars

Additionally, in a pilot study involving 16 ultra-marathon runners (14 males and 2 females; ~31–62 years), the daily consumption of a novel protein bar comprising carbohydrates and whey protein in a 1:1 ratio (2 bars per day) for 2 months resulted in a significant increase in erythrocyte GSH concentration, accompanied by decreased plasma lipid peroxidation and protein carbonylation, thereby enhancing the overall redox profile [117]. Furthermore, in a recent clinical trial, the impact of the daily consumption of an antioxidant-rich bar (1 bar daily) for 4 weeks was investigated in 40 male long-distance runners (20–30 years) [131]. More specifically, the individuals were randomly assigned to a study group consuming a bar with 4-fold higher antioxidant potential and 6-fold higher polyphenol content than a conventional bar or to a control group consuming the conventional bar daily [131]. According to the results, the daily consumption of the antioxidant-rich bar caused a significant trend toward increased plasma GPx activity (*p* = 0.082), as well as a significant decrease in total oxidative status (TOS) and an increase in plasma TAS compared with the baseline [131].

The human nutritional intervention studies and their GSH-related outcomes are summarized in Table 1.

### 4.2. Fasting Protocols and Redox Adaptations

#### 4.2.1. Definition and Mechanistic Basis of Fasting

Fasting refers to a dietary regimen characterized by voluntary abstinence from caloric intake, food, or fluids within a specific time period [132]. Established fasting protocols include time-restricted feeding, where food consumption is limited to predefined eating windows; the 5:2 diet, which involves 5 days of habitual food intake intermixed with 2 non-consecutive fasting days per week; alternate-day fasting, which comprises alternating fasting and feeding days; and religious fasting, which represents one of the oldest forms of periodic dietary restriction [133].

Despite receiving considerable attention as an alternative approach for weight loss and body composition management, growing evidence supports the crucial role of fasting in metabolic, hormonal, and molecular adaptations that dictate energy metabolism, redox homeostasis, inflammation, and cellular resilience [134,135,136,137]. From a biochemical perspective, fasting triggers depletion of circulating glucose and progressive exhaustion of glycogen stored in the liver, activating a “metabolic switch” from glucose utilization to fatty acid oxidation and ketone body production as the primary energy source [138]. This shift promotes metabolic flexibility, enabling efficient exploitation of both fuel sources, thus improving glycemic control, insulin sensitivity, and mobilization of stored adipose tissue for energy production [139,140]. Ketone bodies serve as an efficient energy source for the brain and act as key signaling molecules that regulate gene expression closely related to inflammation, oxidative stress, mitochondrial function, autophagy, and longevity [141].

With respect to the impact of fasting on redox homeostasis, its beneficial effects appear to be mediated, at least in part, through hormesis [142]. Based on this, the mild oxidative stress induced during the fasting window acts as an adaptive stimulus, activating the upregulation of antioxidant defense mechanisms [142,143]. A central modulator of this adaptive response is the transcription factor nuclear factor erythroid-derived 2-like 2 (Nrf2)/Kelch-like ECH-associated protein 1 (Keap1) signaling pathway, the major regulator of antioxidant gene expression [144]. Under homeostatic conditions, Nrf2 is sequestered by the repressor protein Keap1, targeted for constitutive polyubiquitination and proteasomal degradation in the cytosol [145]. This results in low protein levels of Nrf2, which, however, are crucial for ensuring the basal expression of its target genes [146]. Upon oxidative or electrophilic stress conditions, the oxidation of cysteine residues in Keap1 induces the dissociation of Nrf2, enabling its translocation into the nucleus [147]. There, it heterodimerizes with small musculoaponeurotic fibrosarcoma (sMaf) proteins and binds to antioxidant response elements (AREs), initiating the transcription of cytoprotective genes [144].

The proteins encoded by Nrf2-regulated genes are associated with the detoxification of ROS and xenobiotics, the regulation of the GSH antioxidant system, the regeneration of NADPH, and the metabolism of heme and iron [148]. GSH homeostasis is controlled by Nrf2 through the regulation of the two subunits of the glutamate–cysteine ligase complex (GCLC and GCLM) and GR [149]. Furthermore, Nrf2 coordinates the gene expression of crucial antioxidant and phase II detoxifying enzymes, including glutathione peroxidase 2 (GPx2) and glutathione S-transferases (GSTs) [150].

#### 4.2.2. Fasting Protocols and GSH Metabolism

Consistent with these mechanistic insights, human intervention studies demonstrate that different forms of fasting may favorably modulate GSH metabolism and improve redox status. Religious fasting practices, defined by recurring periods of voluntary abstinence from food and, in some cases, water, represent the most thoroughly studied human models for evaluating redox adaptations. Although varying in duration, dietary restrictions, and cultural context, their long-standing tradition provides a robust framework for understanding human redox responses during periods of metabolic stress.

Ramadan fasting constitutes a relevant paradigm, characterized by dry fasting from sunrise to sunset, approximately 12–18 h daily, though evidence from healthy populations is not entirely consistent. For instance, a prospective observational cohort study investigated the impact of 28-day Ramadan fasting, involving complete abstinence from food and water for 16 h daily, on blood redox status in 14 healthy individuals (9 males and 5 females; 25–58 years) [151]. This study reported metabolic benefits, with decreased serum glucose and triglycerides by day 28, yet the effects on redox status were limited to decreased lipid peroxidation in erythrocytes, with no changes in erythrocyte GSH concentration or GPx activity [151]. In contrast, more pronounced effects were observed in a recent prospective cohort study evaluating 21–29 days of Ramadan fasting, during which participants abstained from food and water for 14 h per day, on metabolic health and antioxidant defenses in 62 healthy women, equally allocated to pre-menopausal (21–42 years) and post-menopausal (43–68 years) groups [152]. This intervention significantly improved metabolic status in both groups, as evidenced by favorable changes in anthropometric indicators and inflammatory and oxidative stress markers [152]. Specifically, blood GPx activity increased in both groups, accompanied by elevated SOD activity, collectively underscoring the positive impact of Ramadan fasting on redox balance [152].

While effects in healthy populations vary according to baseline physiological characteristics and oxidative burden, individuals with underlying health conditions appear to exhibit more clinically relevant responses. In a controlled intervention trial of 40 healthy individuals and 40 hypertensive patients (males and females; ~55 years), 4-week Ramadan fasting resulted in elevated erythrocyte GSH levels immediately after the fasting period in both experimental groups, along with reduced lipid peroxidation byproducts, indicating a beneficial effect on redox homeostasis [153]. Notably, GSH levels remained elevated 6 weeks post-fasting in both groups, suggesting sustained redox improvement that extended beyond the fasting period [153]. Furthermore, in a prospective study involving 27 women (18–40 years) with polycystic ovary syndrome (PCOS), a hormonal disorder related to insulin resistance, dyslipidemia, and hyperandrogenism, a 29-day Ramadan fasting, involving complete abstinence from food and water for 16.5 h daily, significantly increased plasma GSH levels [154]. The absence of substantial alterations in other redox markers indicates that Ramadan fasting may selectively enhance GSH-dependent antioxidant pathways in PCOS [154]. Additionally, in a prospective study including 56 overweight and obese adults (34 males and 22 females) and 6 healthy individuals, one-month Ramadan fasting, entailing around 15 h per day of complete abstinence from food and water, significantly improved anthropometric parameters in overweight/obese subjects compared to healthy individuals, and was followed by upregulation of *NFE2L2* and *SOD2* gene expression [155]. This implies that Ramadan fasting triggers a coordinated adaptive response, enhancing cellular antioxidant defense systems, particularly in individuals with increased adiposity and oxidative burden [155].

Christian Orthodox fasting, typically constituting a plant-based regimen with fixed eating windows rather than complete abstinence from food and water, also provides valuable insights into how different forms of religious fasting influence GSH homeostasis and metabolism. In a comparative clinical trial enrolling 50 vitamin D-deficient, overweight orthodox nuns (30–50 years) and 50 lay women (>18 years), participants followed 16 weeks of Christian Orthodox fasting (16 h daily fast and an 8 h eating window with a plant-based diet including seafood and fish) and standard 16:8 time-restricted eating with a conventional diet, respectively, revealing distinct antioxidant advantages [156]. The monastic cohort exhibited higher plasma total antioxidant capacity compared to lay women, whereas erythrocyte GSH levels were conversely higher in lay women [156].

Modern fasting protocols also show heterogeneous effects on GSH regulation and function. A 10-day medically supervised fasting protocol with caloric restriction (250 kcal daily) in 109 participants (41 males and 68 females; 18–70 years) resulted in improved blood redox status, demonstrated by increased total antioxidant capacity and reduced lipid peroxidation in plasma [157]. This prospective observational study represents the largest cohort examined for fasting effects on redox status to date and documented substantial metabolic improvements, including reductions in body weight and waist circumference, improved lipid profile and glycemic control, and enhanced physical and emotional well-being. Nevertheless, no significant effects were observed on erythrocyte GSH concentration or GSH-dependent enzymes, suggesting that the redox benefits induced by this fasting regimen are mediated predominantly through mechanisms independent of direct GSH modulation [157]. Furthermore, a 3-month randomized study examined the combined effects of TRE and physical activity on inflammation and redox status in 25 healthy individuals (12 males and 13 females; 21–57 years and 21–58 years, respectively), with 7 participants allocated to the TRE group following a typical 16:8 pattern and 18 participants to the control group following a balanced diet [158]. Nevertheless, no significant differences were observed between groups in salivary GSH concentration, which also remained stable across all physical activity levels, indicating that TRE alone does not alter GSH homeostasis in healthy individuals [158]. Contrariwise, a robust anti-inflammatory effect was observed, as interleukin-6 (IL-6) concentrations were significantly lower in the TRE group compared with the control group [158]. Finally, a metabolomic study involving 4 healthy individuals (3 males and 1 female; 29–30 years) investigated the impact of a 58 h fasting period, with consumption limited to calorie-free beverages, on the blood metabolome [159]. Fasting was accompanied by enhanced activity of the pentose phosphate pathway (PPP), indicative of increased NADPH production, which is essential for the regeneration of GSH from GSSG [159]. Despite this, whole blood GSSG concentration remained stable throughout the fasting period, while concentrations of ophthalmic acid, a GSH analog and proposed biomarker of GSH turnover, increased significantly [159]. Altogether, these findings support the notion that fasting promotes antioxidant adaptations and maintenance of redox balance through mechanisms that support GSH recycling and utilization, rather than through marked changes in the GSH pool [159].

The principal characteristics and GSH-related outcomes of the fasting interventions discussed above are summarized in Table 2.

## 5. Concluding Remarks and Future Perspectives

The expanding gap between lifespan and healthspan constitutes a critical public health burden, with disturbances of normal metabolic function emerging as the primary driver of chronic NCDs. The purpose of this review was to highlight the importance of redox homeostasis in maintaining and improving metabolic health, placing GSH at the forefront of this endeavor. Indeed, GSH serves as the master regulator of redox balance, participating in antioxidant defense, xenobiotic detoxification, redox signaling, and metabolic regulation. Perturbations in circulating GSH levels are linked to metabolic dysfunction in obesity, type 2 diabetes mellitus, and cardiovascular diseases. From this perspective, enhancement of GSH metabolism through dietary and lifestyle interventions represents a promising non-pharmacological strategy for improving metabolic health, particularly in later life, and for narrowing the gap between lifespan and healthspan.

**Figure 1:**
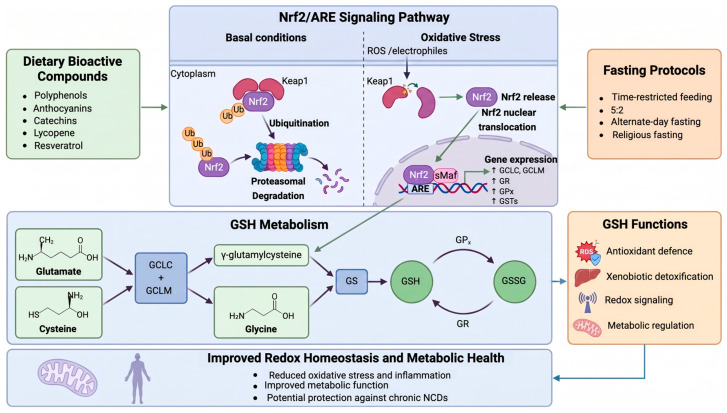
Proposed mechanisms linking dietary bioactive constituents and fasting protocols with GSH homeostasis, redox balance, and metabolic health. Created in BioRender. Vardakas, P. (2026) https://BioRender.com/petfk49.

Bioactive compounds with antioxidant properties, present in various food products, including aromatic plants, herbs, fruits, and vegetables, contribute directly to the preservation of redox status by neutralizing excess ROS. Nevertheless, their primary effects in vivo appear to be exerted via activation of the Nrf2/ARE signaling pathway. Through this mechanism, the expression of crucial genes associated with GSH synthesis, utilization, and regeneration is upregulated. Similarly, fasting activates Nrf2 via short-term, low-grade oxidative stress, triggering hormetic mechanisms. The proposed mechanisms linking dietary bioactive compounds and fasting with GSH homeostasis, redox balance, and metabolic health are illustrated in Figure 1. At the same time, heterogeneity in fasting protocols leads to inconsistent effects on GSH, with specific studies reporting no alterations in its levels or related enzymatic activities, despite overall redox status being improved in most cases. Variability in GSH responses is modulated by several factors, including genetic background, sex, age, gut microbiota composition, nutritional and health status, and metabolic phenotype, underscoring the need for precision dietary strategies. Notably, the beneficial effects of both approaches appear to be more pronounced in individuals exposed to increased oxidative stress or manifest metabolic dysfunction. Nevertheless, current evidence remains limited by the relatively small number of rigorously controlled clinical trials, the heterogeneity of intervention protocols, and the variability in biomarkers employed to assess GSH homeostasis and metabolism.

To the best of our knowledge, this is the first narrative review to integrate clinical evidence from human studies on both bioactive-rich food products and fasting protocols from the perspective of GSH modulation and redox-driven metabolic health. Recent insightful reviews have either focused broadly on GSH biology and its therapeutic potential [160,161,162,163] or have investigated dietary antioxidants [164,165,166,167,168] and fasting protocols [136,137,169,170] in relation to oxidative stress and metabolic health separately. By collating and critically appraising intervention studies across healthy, athletic, metabolically impaired, and chronically diseased cohorts, this work describes context-dependent patterns whereby GSH responses and the subsequent redox benefits seem to be more pronounced in individuals with elevated oxidative burden or metabolic dysfunction.

Future research initiatives could focus on: (a) mechanistic investigations designed to elucidate the molecular pathways that link Nrf2 activation, GSH metabolism, and metabolic signaling; (b) large-scale, long-term randomized, controlled trials examining the efficacy of dietary and lifestyle interventions aimed at enhancing GSH for the prevention of NCDs; and (c) integrated approaches incorporating the consumption of bioactive-rich foods, fasting, and moderate physical activity, tailored to individuals to account for interindividual variability in redox status and metabolism. As scientific interest moves from prolonging lifespan toward extending healthspan, strategies that enhance redox status, and particularly GSH homeostasis may represent promising interventions against age-related NCDs.

## Figures and Tables

**Table 1:** Bioactive-rich foods and GSH-related redox adaptations in human studies.

Food/Product Type	Intervention Characteristics	Population (n, Sex, Age)	Health Status	GSH-Related Outcome	Reference
Green tea (extract)	6-week CrossFit program; 500 mg/day green tea extract vs. placebo; two exercise trials pre- and post-intervention	16 males (22–23 years)	Healthy, physically active	No significant changes in whole blood GSH concentration, erythrocyte GPx or GR activities	[112]
Green tea (brew)	8 weeks; 4 cups/day green tea vs. 4 cups/day water	35 individuals (8 males and 27 females; 25–63 years)	Obese, metabolic syndrome	Increased whole blood GSH concentration; no significant changes in serum GPx activity	[113]
Green tea epicatechin (ex vivo)	Concentration-dependent incubation of blood samples with (−)-epicatechin	Healthy and hypertensive individuals (n, sex and age not specified)	Healthy and hypertensive	Increased erythrocyte GSH concentration in both groups with stronger effects in hypertensive subjects	[114]
Tomato juice	18 days; 240 g/day tomato juice	25 males (10–11 years)	Healthy	No significant change in whole blood GSH concentration	[116]
Tomato juice	2 months; tomato juice provided in amounts isocaloric with the habitual carbohydrate supplement	15 ultra-marathon runners (13 males and 2 females; 36–53 years)	Healthy, endurance trained	No significant change in erythrocyte GSH concentration	[117]
Tomato juice	20 days; 330 mL/day tomato juice vs. usual lifestyle	64 females (20–30 years)	Overweight/obese	Increased erythrocyte GPx activity	[118]
Tomato juice and calorie restriction	60 days; 1200 kcal/day regimen ± 100 mL/day tomato juice	61 individuals (33 males and 28 females; 8–13 years)	Obese, NAFLD	Increased serum GSH concentration, decreased serum GSSG concentration, increased GSH/GSSG ratio	[119]
Pomegranate juice	4 weeks; 250 mL/day	13 individuals (10 males and 3 females; >60 years)	Healthy	Increased plasma GPx activity; no change in plasma GSH concentration	[121]
Pomegranate juice	15 days; 500 mL/day	14 individuals (8 males and 6 females; 30–37 years)	Healthy	Increased erythrocyte GSH concentration	[122]
Pomegranate juice	Acute high-dose: 250 mL 3×/day for 48 h and 500 mL 60 min pre-exercise (total 1.25 L), repeated for two training sessions	9 males (20–22 years)	Healthy, elite weightlifters	Increased plasma GPx activity shortly post-exercise and faster return to baseline over 48 h and 10 days	[123]
Pomegranate juice	6 weeks; 200 mL/day vs. usual lifestyle	39 individuals (4 males and 35 females; ~47–67 vs. ~42–66 years in intervention vs. control)	Osteoarthritis	Increased serum GPx activity	[124]
Grape juice (conventional and organic)	Crossover; 400 mL/day conventional grape juice, organic grape juice, or water, with 14-day washout periods; measurements pre and 1 h post-ingestion	30 individuals (9 males and 21 females; ~20–38 and ~21–36 years)	Healthy	Increased erythrocyte GSH concentration and GPx activity with both conventional and organic juice vs. baseline and water	[126]
Grape seed extract	8 weeks; 300 mg twice daily vs. placebo	40 female volleyball players (~15–29 years)	Healthy, physically active	Increased plasma GSH concentration	[127]
Red grape skin polyphenolic extract	6 weeks; 390 mg three times daily vs. control during interval swimming training	14 male physical education students (21–22 years)	Healthy, trained	No significant changes in erythrocyte GSH concentration, GPx or GR activities	[128]
Brazil nuts	3 months; 1 Brazil nut/day	81 individuals (55 males and 26 females; ~37–67 years)	Chronic kidney disease, hemodialysis	Increased erythrocyte and plasma selenium; increased erythrocyte GPx activity; values normalized into reference range	[129]
Brazil nuts	8 weeks; 1 Brazil nut/day (~290 μg selenium/day)	37 females (28–41 years)	Severe obesity	Increased erythrocyte GPx activity; higher erythrocyte and plasma selenium	[130]
Protein bar (carbohydrate–whey, 1:1)	2 months; 2 bars/day	16 ultra-marathon runners (14 males and 2 females; ~31–62 years)	Healthy, endurance trained	Increased erythrocyte GSH concentration	[117]
Antioxidant-rich bar	4 weeks; 1 bar/day with 4-fold higher antioxidant potential and 6-fold higher polyphenols vs. conventional bar	40 male long-distance runners (20–30 years)	Healthy, endurance trained	Trend toward increased plasma GPx activity (*p* = 0.082)	[131]

**Table 2:** Fasting protocols and GSH-related redox adaptations in human studies.

Fasting Protocol Type	Fasting Protocol Characteristics	Population (n, Sex, Age)	Health Status	GSH-Related Outcome	Reference
Ramadan dry fasting	28 days, 16 h/day, complete abstinence from food and water	14 individuals (9 males and 5 females; 25–58 years)	Healthy	No significant changes in erythrocyte GSH concentration or GPx activity	[151]
Ramadan dry fasting	21–29 days, 14 h/day, complete abstinence from food and water	62 females (31 pre-menopausal, 21–42 years; 31 post-menopausal, 43–68 years)	Healthy	Increased blood GPx activity in both groups	[152]
Ramadan dry fasting	4 weeks, sunrise–sunset, complete abstinence from food and water	80 individuals (40 healthy and 40 hypertensive, males and females; ~55 years)	Healthy and hypertensive	Increased erythrocyte GSH concentration in both groups (sustained 6 weeks post-fasting)	[153]
Ramadan dry fasting	29 days, ~16.5 h/day, complete abstinence from food and water	27 females (18–40 years)	PCOS	Increased plasma GSH concentration	[154]
Ramadan dry fasting	1 month, ~15 h/day, complete abstinence from food and water	56 overweight/obese individuals (34 males and 22 females) and 6 healthy individuals (males and females)	Overweight/obese and healthy	Increased *NFE2L2* gene expression in overweight/obese individuals compared to healthy individuals	[155]
Christian Orthodox fasting vs. 16:8 time-restricted eating (TRE)	16 weeks, 16 h/day, plant-based diet with seafood and fish vs. 16 weeks, 16 h/day, conventional diet	50 nuns (30–50 years) vs. 50 lay women (>18 years)	Overweight, vitamin D-deficient vs. healthy	Increased erythrocyte GSH concentration in lay women compared to nuns	[156]
10-day medically supervised fasting	10 days, consumption of 250 kcal/day	109 individuals (41 males and 68 females; 18–70 years)	Healthy	No significant changes in erythrocyte GSH concentration, GPx and GR activities	[157]
16:8 time-restricted eating (TRE) and physical activity	3 months, 16 h/day	25 individuals (12 males and 13 females; 21–57 years and 21–58 years, respectively)	Healthy	No significant changes in salivary GSH concentration	[158]
58 h fasting	58 h, consumption of calorie-free beverages	4 adults (3 males and 1 female; 29–30 years)	Healthy	No significant changes in whole blood GSSG concentration; increased plasma PPP-related metabolites and whole blood ophthalmic acid concentration	[159]

## Data Availability

No new data were created or analyzed in this study. Data sharing is not applicable to this article.
